# Genetic diversity of the *Plasmodium falciparum* GTP-cyclohydrolase 1, dihydrofolate reductase and dihydropteroate synthetase genes reveals new insights into sulfadoxine-pyrimethamine antimalarial drug resistance

**DOI:** 10.1371/journal.pgen.1009268

**Published:** 2020-12-31

**Authors:** Anna Turkiewicz, Emilia Manko, Colin J. Sutherland, Ernest Diez Benavente, Susana Campino, Taane G. Clark

**Affiliations:** 1 Department of Infection Biology, London School of Hygiene and Tropical Medicine, London, United Kingdom; 2 Department of Infectious Disease Epidemiology, Faculty of Epidemiology and Population Health, London School of Hygiene and Tropical Medicine, London, United Kingdom; University of Pennsylvania, UNITED STATES

## Abstract

*Plasmodium falciparum* parasites resistant to antimalarial treatments have hindered malaria disease control. Sulfadoxine-pyrimethamine (SP) was used globally as a first-line treatment for malaria after wide-spread resistance to chloroquine emerged and, although replaced by artemisinin combinations, is currently used as intermittent preventive treatment of malaria in pregnancy and in young children as part of seasonal malaria chemoprophylaxis in sub-Saharan Africa. The emergence of SP-resistant parasites has been predominantly driven by cumulative build-up of mutations in the dihydrofolate reductase (*pfdhfr*) and dihydropteroate synthetase (*pfdhps*) genes, but additional amplifications in the folate pathway rate-limiting *pfgch1* gene and promoter, have recently been described. However, the genetic make-up and prevalence of those amplifications is not fully understood. We analyse the whole genome sequence data of 4,134 *P. falciparum* isolates across 29 malaria endemic countries, and reveal that the *pfgch1* gene and promoter amplifications have at least ten different forms, occurring collectively in 23% and 34% in Southeast Asian and African isolates, respectively. Amplifications are more likely to be present in isolates with a greater accumulation of *pfdhfr* and *pfdhps* substitutions (median of 1 additional mutations; P<0.00001), and there was evidence that the frequency of *pfgch1* variants may be increasing in some African populations, presumably under the pressure of SP for chemoprophylaxis and anti-folate containing antibiotics used for the treatment of bacterial infections. The selection of *P. falciparum* with *pfgch1* amplifications may enhance the fitness of parasites with *pfdhfr* and *pfdhps* substitutions, potentially threatening the efficacy of this regimen for prevention of malaria in vulnerable groups. Our work describes new *pfgch1* amplifications that can be used to inform the surveillance of SP drug resistance, its prophylactic use, and future experimental work to understand functional mechanisms.

## Author summary

Malaria causes approximately 435,000 deaths per year, concentrated in sub-Saharan Africa and among children under the age of five years. Global efforts to control and eliminate malaria are hampered by the emergence of *Plasmodium falciparum* malaria parasites resistant to currently available antimalarial drugs. Sulfadoxine-pyrimethamine (SP) was used globally as a first-line treatment for malaria and, although replaced by artemisinin combinations, is still used for the prevention of malarial disease in vulnerable groups (e.g. pregnant women). SP resistance is caused by mutations in the *P. falciparum* parasite genes *pfdhfr* and *pfdhps*, but recently novel structural variants in and around the *pfgch1* gene have been described. By analysing genome sequence data of 4,134 *P. falciparum* across 29 malaria endemic countries, we establish there are at least ten different *pfgch1* structural variants, existing in the presence of *pfdhfr* and *pfdhps* mutations, and occur increasingly and at high frequency in some Southeast Asian and African populations. These *pfgch1* structural variants may enhance the survival of those parasites with *pfdhfr* and *pfdhps* substitutions, potentially threatening the efficacy of SP for prevention of malaria in vulnerable groups. Therefore it is important that they are monitored in molecular surveillance studies. Our work will assist epidemiological studies, laboratory and surveillance activities looking at the diversity and role of *pfgch1* structural variants and their prevalence across malaria endemic regions, especially in countries using SP regimens.

## Introduction

The *Plasmodium falciparum* parasite inflicts high morbidity and mortality on human populations in malaria endemic regions, especially in sub-Saharan Africa. To inform control measures, investigations of *P. falciparum* adaptation for host immune evasion, antimalarial drug resistance and other important biological mechanisms have focused on analyses of genome-wide polymorphisms [1, 2]. A number of studies have revealed SNPs and structural variants (e.g. duplications, amplifications or copy number variants) linked to antimalarial drugs, such as chloroquine, sulfadoxine-pyrimethamine (SP) and artemisinin [3]. SP is a combination drug which inhibits the dihydrofolate reductase (*dhfr*) and dihydropteroate synthetase (*dhps*) enzymes in the folate pathway of the parasite, and is widely used as intermittent preventive treatment of malaria in pregnancy (IPTp) and in infants as part of seasonal malaria chemoprophylaxis (SMC) in sub-Saharan Africa. SP became a first-line treatment for malaria after widespread resistance to chloroquine, but was replaced by artemisinin combination therapies for uncomplicated cases with the increasing prevalence of *P. falciparum* mutant alleles that confer the parasite resistance to pyrimethamine in *pfdhfr* (N51I, C59R, S108N, I164L) and to sulfadoxine in *pfdhps* (I431V, S436A/F, A437G, K540E/N, A581G, A613S/T) genes [4, 5]. Sequential accumulation of the several point mutations leads to increased levels of resistance by reducing binding affinity of the drug to the folate pathway enzymes *dhps* and *dhfr* [6].

Evidence across many countries has shown that longer term usage of SP leads to a greater risk of resistance haplotypes in *pfdhps* and *pfdhfr* genes [1]. The increased prevalence in parasites with resistant haplotypes is due to selection by drug pressure from the use of SP for IPTp and SMC as well as the use of anti-folate containing antibiotics. Fixation of some of the key SP-resistant mutations in the parasite population may occur, despite discontinuation of SP as the first-line treatment for more than a decade [7], and understanding these genetics dynamics is crucial for malarial disease control. Genomic analyses of the malaria parasites have revealed copy number variations of the GTP cyclohydrolase I gene (*pfgch1*), which encodes the first and the rate-limiting enzyme in the folate biosynthesis pathway. Increased copy number of *pfgch1* has been linked to SP resistance in Southeast Asia [8], with a direct association to *pfdhfr* and *pfdhps* alleles [9]. Similarly, a *pfgch1* promoter copy number variation (amplification) in Malawian parasites with quintuple mutations (I51-R59-N108-G437-E540) has been identified, which differs from the whole gene amplification found in Southeast Asia [1, 10]. The multiple copies of *pfgch1* are thought to compensate for the putatively fitness-reducing mutations in *pfdhfr* and *pfdhps* by providing higher concentrations of upstream substrates in the folate-biosynthetic pathway [10].

Here we investigate the genetic diversity in the *pfgch1*, *pfdhfr* and *pfdhps* genes across 4,134 *P. falciparum* isolates from 29 malaria endemic populations. Using both long and short sequence data, we reveal there are multiple forms of *pfgch1* promoter and gene copy number variations, whose frequencies are heterogeneously distributed geographically, and linked to *pfdhfr* and *pfdhps* haplotypes. Worryingly, we reveal an overall trend towards an increased prevalence of the known *pfdhfr* and *pfdhps* resistant markers, which may be attributed to SP drug pressures, and that the presence of amplified *pfgch1* is exacerbating the existing problem and may be assisting with maintaining these resistant parasites and the evolution of new mutants.

## Materials and methods

### Short read sequencing data

Publicly available raw sequence data were downloaded for *P. falciparum* field isolates (n = 6,236; 30 countries; from 2001 to 2015) from the European Nucleotide Archive (study accessions ERP000190 and ERP000199). These data include Illumina raw sequences from the MalariaGEN Community Project [11]. The data were aligned to the 3D7 strain reference genome (v3.1) using *bwa-mem* software (default parameters, except –c 100 –T 50). Multiplicity of infection (MOI) was calculated using *estMOI* software [12] and the threshold was obtained based on previous work [13]. Samples with MOI >1 (estMOI > 30%) and overall genomic coverage <10-fold were removed from the dataset, leading to 5,280 isolates used for further analysis. SNPs and small indels were called using the *samtools* software suite, and those with a minor allele frequency >1% in drug resistance loci retained. The final dataset contained 4,134 samples (see pathogenseq.lshtm.ac.uk for a list of ENA accession numbers) without missing or mixed SNP genotype calls in *pfdhfr*, *pfdhps* and *pfgch1*. Large duplications were called by applying *Delly* software (v0.8.1) with default settings, and their breakpoints inferred using split-reads. All structural variants of low-quality (supporting paired-end calls < 3 or average mapping quality < 20) and >100kbp in length were removed, with 1,323 potential events located in the *pfgch1* gene locus or its promoter region. All isolates with multiple calls in the region of interest were also removed and 1,273 remaining duplications were clustered (start and end breakpoints ± 50) to create 10 unique events. Coverage at a chromosome, regional and locus level was calculated from the alignment files for each isolate, and used to infer a *pfgch1* gene and promoter copy number in those isolates with amplifications. The copy number estimation involved normalisation using a median coverage of isolates with no duplications. African samples were classified into geographical regions based on an established grouping [14].

### Long read sequencing data

Four laboratory parasite strains (K1 Thailand, D10 Papua New Guinea, NF54 Africa, T996 Thailand) were cultured under standard conditions [15] at the LSHTM and DNA was extracted using the Genomic-tip 100/G kit (QIAGEN) according to its protocol. The DNA was sequenced on the PacBio RSII long read technology. Chromosome-wide assemblies of PacBio sequence data were also available for a further five laboratory strains (7G8 Brazil, DD2 Indochina, GB4 Ghana, HB3 Honduras, IT Brazil) and nine field isolates (GN01 Guinea, SN01 Senegal, CD01 Congo, ML01 Mali, GA01 Gabon, KE01 Kenya, SD01 Sudan, and KH01 and KH02 Cambodia) (accession number ERP009847) [16]. Raw PacBio sequencing data were analysed in the SMRT Portal software using the Hierarchical Genome Assembly Process (HGAP3) pipeline, resulting in corrected long reads for each sample, and total genome sizes of 24Mbp. Corrected reads were aligned to the 3D7 reference genome (v3.1) using *NGMLR* (v0.2.7) software with default settings. Structural variants were identified using *Sniffles* (v1.0.11) software with default parameters. Manual verification was performed through alignment of candidate regions with the *Mauve* software [17]. All of the PacBio strains with reported structural variants in the *pfgch1* locus or its promoter region were used to validate any possible putative duplications found in the Illumina short read analysis, estimate breakpoints, and distinguish different types of amplifications identified.

### Statistical analysis

The calculation of the frequencies and visualisation of data were performed in *R* software using multiple packages (e.g. *dplyr*, *tidyr*, *tibble*, *gggenes*, *rworldmap* and *tidyverse*). Wilcoxon tests were used to compare the differences in mutation frequencies between structural variant and geographical groups. Genotypic resistance was established for chloroquine using *pfcrt* (C72S, M74I, N75D/E, K76T, A144F, L148I, I194T, A220S, Q271E, A326D/S, I356L/T and R371I) and *pfmdr1* (N86Y) mutations [18–20], and for artemisinin using *pfkelch13* mutations (F446I, Y493H, P574L, R539T, and C580Y) [21]. Pearson correlation coefficients and allelic associations (e.g. linkage disequilibrium *r*^2^) were calculated between SNPs and amplifications using the *polycor* and *LDcorSV* packages.

## Results

### Global frequencies of *pfdhfr* and *pfdhps* genotypes

High quality SNPs for genes in the folate pathway were characterised from Illumina whole genome sequencing data across 4,134 isolates representing West Africa (n = 1,254, 10 countries), Central Africa (n = 337, 2 countries), East Africa (n = 270, 3 countries), Southern Africa (n = 234, 3 countries), Horn of Africa (n = 22, 1 country), South(east) Asia (n = 1,890, 6 countries), Oceania (n = 95, 2 countries), and South America (n = 32, 2 countries). There were thirteen common non-synonymous mutations (minor allele frequency > 1%) in *pfdhfr* (N51I, C59R, S108N, I164L and S306F) and *pfdhps* (I431V, S436A, S436F, A437G, K540E, K540N, A581G and A613S), which collectively formed 38 common genotypes across all of the studied regions (frequency of >1% for any of the region) (Table 1; S1 Table).

**Table 1:** The frequency (%) of *pfdhfr*/*pfdhps* genotypes by region across the 4,134 *P. falciparum* isolates.

Genotypes *	No. muts	West Africa (n = 1254)	Central Africa (n = 337)	East Africa (n = 270)	Southern Africa (n = 234)	Horn of Africa (n = 22)	South(east) Asia (n = 1890)	Oceania (n = 95)	South America (n = 32)
NCSIS-ISAKAA	WT	1.8	-	-	1.3	-	0.4	-	-
NCSIS-IS**G**KAA	1	5.5	-	-	-	-	-	-	-
NCSIS-I**A**AKAA	1	6.1	-	-	-	-	-	-	-
NC**N**IS-ISAKAA	1	-	-	-	-	-	-	-	**56.3**
NCSIS-I**AG**KAA	2	3.4	-	-	-	-	-	-	-
N**RN**IS-ISAKAA	2	-	-	-	-	-	1.5	9.5	-
**I**C**N**IS-ISAKAA	2	-	-	1.5	-	-	-	-	9.4
**I**C**N**IS-IS**G**KAA	3	0.7	9.8	-	-	-	-	-	-
N**RN**IS-IS**G**KAA	3	3.1	-	-	-	-	0.8	-	-
**IRN**IS-ISAKAA	3	5.7	2.1	5.2	-	-	4.1	-	-
N**RN**IS-I**A**AKAA	3	1.3	-	-	-	-	0.2	-	-
N**RN**I**F**-ISAKAA	3	-	-	-	-	-	-	**48.4**	-
N**RN**IS-IS**GE**AA	4	-	-	2.2	1.7	-	0.3	4.2	-
N**RN**IS-I**AG**KAA	4	1.9	-	-	-	-	0.7	-	-
N**RN**I**F**-IS**G**KAA	4	-	-	-	-	-	-	7.4	-
**I**C**N**IS-IS**GE**AA	4	-	1.2	5.2	-	-	-	-	-
**IRN**IS-IS**G**KAA	4	**30.1**	**59.3**	-	3.4	-	3.4	-	-
**IRN**IS-I**A**AKAA	4	14.6	3.3	2.2	-	-	0.5	-	-
N**RN**IS-I**AGE**AA	5	-	-	-	-	-	1.7	-	-
N**RN**I**F**-IS**GE**AA	5	-	-	-	-	-	-	**28.4**	-
**I**C**N**IS-IS**GEG**A	5	-	-	-	-	-	-	-	9.4
**IRN**IS-IS**G**K**G**A	5	-	-	-	1.7	-	0.5	-	-
**IRN**IS-IS**GE**AA	5	0.9	1.8	**58.1**	**89.3**	**86.4**	3.2	-	-
**IRN**IS-I**AG**KAA	5	**15.8**	5.0	-	-	-	4.2	-	-
N**RNL**S-IS**GEG**A	6	-	-	-	-	-	1.0	-	-
N**RNL**S-I**AGE**AA	6	-	-	-	-	-	1.7	-	-
**IRN**IS-IS**GNG**A	6	-	-	-	-	-	4.1	-	-
**IRN**IS-IS**GEG**A	6	-	1.2	**20.0**	-	-	3.8	-	-
**IRN**IS-I**AG**KA**S**	6	3.0	1.8	-	-	-	-	-	-
**IRN**IS-I**AGE**AA	6	-	-	-	-	-	9.0	-	-
**IRN**IS-**VAG**KAA	6	-	4.2	-	-	-	-	-	-
**IRNL**S-IS**GE**AA	6	-	-	-	-	-	1.3	-	-
**IRNL**S-I**FG**KAA	6	-	-	1.1	-	-	-	-	-
**IRNL**S-IS**GNG**A	7	-	-	-	-	-	14.1	-	-
**IRNL**S-IS**GEG**A	7	-	-	-	-	-	**26.7**	-	-
**IRNL**S-I**AGE**AA	7	-	-	-	-	-	10.5	-	-
**IRNL**S-I**FGE**A**S**	8	-	-	-	-	-	1.4	-	-
**IRN**IS-**VAG**K**GS**	8	1.7	6.8	-	-	-	-	-	-

WT wild-type; No. muts is the number of mutations deviating from wild-type;

* based on *pfdhfr* (N51I, C59R, S108N, I164L and S306F) and *pfdhps* (I431V, S436A/S436F, A437G, K540E/K540N, A581G and A613S; frequencies greater than 15% are bolded)

A quadruple *pfdhfr*/*pfdhps* mutant genotype (*pfdhfr* N51I/C59R/S108N and *pfdhps* A437G; **IRN**IS-IS**G**KAA) was frequent in Central Africa (59.3%) and West Africa (30.1%), consistent with other studies [22, 23]. Furthermore, the octuple mutant (**IRN**IS-**VAG**K**GS**) with unique mutations in *pfdhps* I431V and A613S was characterised primarily in Benin (26.2%) and Cameroon (14.6%) (S2 Table) [5]. The *pfdhps* I431V is generally thought of as West African because it was first described in Nigeria, but in our limited set of isolates from that country (n = 19) it was not detected. Other prevalent genotypes in West Africa were the quadruple (**IRN**IS-I**A**AKAA) (14.6%) and quintuple (**IRN**IS-I**AG**KAA) (15.8%) mutant genotypes. The quintuple mutant genotype (**IRN**IS-IS**GE**AA) was predominant in Southern Africa (89.3%), and eastern regions of the continent (Horn of Africa, 86.4%; East Africa, 58.1%). Furthermore, in eastern regions, the **IRN**IS-IS**GEG**A genotype mutant was reported (overall 20.0%; Tanzania, 25.0%) (S2 Table). The most prevalent genotypes in Southeast Asia were the septuple mutants (**IRNL**S-IS**GEG**A, 26.7%; **IRNL**S-IS**GNG**A, 14.1%) which include the regional specific *pfdhfr* I164L mutation, and differ between themselves by the *pfdhps* K540E/N SNP. The **IRNL**S-IS**GEG**A genotype mutant was most frequent in Thailand and Myanmar, while the **IRNL**S-IS**GNG**A was frequent in Cambodia (S2 Table). Two genotypes were prominent in Oceania (N**RN**I**F**-ISAKAA, 48.4%; N**RN**I**F**-IS**GE**AA, 28.4%), which were not prominent in other regions (<0.1%), and contain a regional specific mutation (*pfdhfr* S306F). Lastly, the South American population was characterised by the NC**N**IS-ISAKAA (56.3%) genotype mutant.

### *Pfgch1* amplification types and their global distribution

**Fig 1:**
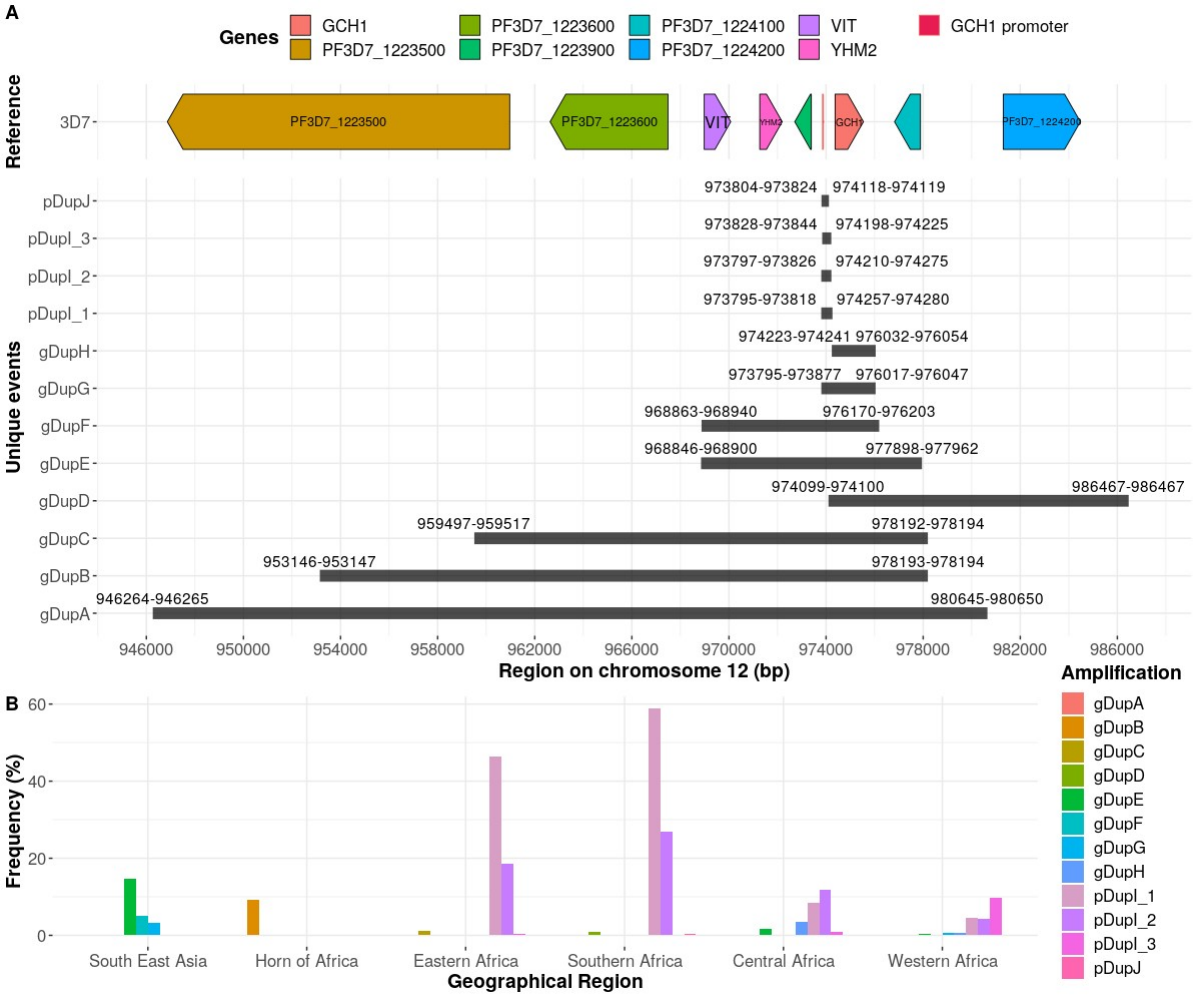
The *pfgch1* amplifications (DupA-J) and their frequencies (%). A. Amplification (Dup) genetic map. B. Barplot of amplification (Dup) frequencies by each geographical region.

Across the 4,134 isolates we identified ten unique amplifications (denoted DupA—DupJ), present in 1,171 (28.3%) samples (Fig 1). Amplifications were in the *pfgch1* gene or promoter regions. The majority (681/1171; 58.2%) involved *pfgch1* promoter only regions (pDupI, pDupJ) and therefore were of short length, and were found exclusively in African populations (Table 2; S3 Table). The *pfgch1* genic amplifications (gDupA-H) were longer in length and had high variability in frequency (Fig 1). Amplifications gDupA (34 kb, Cambodia, n = 3), gDupB (25 kb, Ethiopia, n = 2) and gDupC (19 kb, Cambodia, n = 2; Kenya, n = 3) were less prevalent than others, but longer and included up to six neighbouring genes (*PF3D7_1223500, Pf3D7_1223600, VIT, YHM2, PF3D7_1223900, PF3D7_1224100*). The gDupD amplification (12 kb, Madagascar, n = 2) contained only *pfgch1*, *PF3D7_1224100* and *PF3D7_1224200* loci. The gDupE amplification contained 5 genes (*VIT, YHM2, PF3D7_1223900, pfgch1, PF3D7_124100*) and was the most frequent in Southeast Asia (n = 279, 14.8%; Thailand, Myanmar and Cambodia), but was also present in African countries (n = 10, 0.5%; Ghana, Cameroon, Democratic Republic of Congo). The gDupF (∼7.3 kb) amplification spans four loci and is present in South/Southeast Asia (n = 98, 5.2%; Thailand, Myanmar and Bangladesh) and Ghana (n = 3, 0.6%). The gDupG amplification only involves the modification of the *pfgch1* locus and its promoter, and was mostly observed in Southeast Asian isolates (n = 59, 3.1%; Cambodia, Vietnam and Thailand) and Ghana (n = 8, 1.7%). Additionally, we found 5 unique amplifications suggesting that more variants may exist across regions of interest.

**Table 2:** Distribution (%) of the *pfgch1* amplifications (gene DupA-H; promoter DupI-J).

Region	n	Any	A	B	C	D	E	F *	G **	H ***	I +	I ++	I +++	J
West Africa	1254	**20.3**	-	-	-	-	0.3	0.2	0.7	0.6	4.5	4.3	9.6	0.1
Central Africa	337	**26.4**	-	-	-	-	1.8	-	-	3.6	8.3	**11.9**	0.9	-
East Africa	270	**66.3**	-	-	1.1	-	-	-	-	-	**46.3**	**18.5**	0.4	-
Southern Africa	234	**87.2**	-	-	-	0.9	-	-	-	-	**59.0**	**26.9**	-	0.4
Horn of Africa	22	9.1	-	9.1	-	-	-	-	-	-	-	-	-	-
Southeast Asia	1890	**23.4**	0.2	-	0.1	-	**14.8**	5.2	3.1	0.1	-	-	-	-

* GB4, T996;

** KH02;

*** NF54;

+ KE01;

++ KE01/ML01;

+++ ML01;

DupA—H are gene duplications; DupI (3 types) and DupJ are promoter duplications; (n = 107), Colombia (n = 16) and Peru (n = 22) have no amplifications; frequencies greater than 10% are bolded

Collectively, one of every five Southeast Asian isolates (23.1%) had a gene (gDupE, gDupF or gDupG) amplification, with the highest occurrence in Thailand and Myanmar (38.9% and 34.4% respectively). The gDupH is an amplification of the *pfgch1* locus, but not its promoter, and was almost exclusively observed in African samples (n = 19, 0.9%; Cameroon, n = 12). The gDupF, gDupG and gDupH amplifications have been reported in Thailand [8]. While pDupI and pDupJ amplifications appear exclusively in Africa and consist of the promoter of the *pfgch1* gene (chr. 12: 973,848–973,907; EPD promoter ID: CZT99398_1 [24]). The pDupI (∼400 kb) amplification was discovered in Malawian isolates [1] and occurs with a high frequency in the African continent (West 18.4%, Central 21.1%; East 65.2%, Southern 85.9%). The pDupJ (∼310bp) amplification is a novel modification reported in two isolates.

### Confirming amplifications and their breakpoints using long-read sequences

To assist the refinement of amplification breakpoints and study of the surrounding sequences, we analysed long-read PacBio data for eighteen *P. falciparum* strains, including nine laboratory (K1, D10, NF54, T996, 7G8, DD2, GB4, HB3, IT) and nine field isolates (GN01, SN01, CD01, ML01, GA01, KE01, SD01, KH01 and KH02). Strains GB4 (Ghana) and T996 (Thailand) had a gDupF amplification with similar breakpoints (chr. 12 location: GB4 968,859–976,203; T996 968,862–976,203), but different numbers of copies (Ghana 3; T996 4). The gDupG amplification was identified in the Cambodian field isolate KH02 (chr. 12: 973,794–976,052; 2 copies). The gDupH amplification was confirmed in the NF54 African laboratory strain (chr. 12: 974,231–976,053) and carried four copies of the *pfgch1* gene. Importantly, geographical locations of the PacBio *pfgch1* copy number variants matched those identified in the clinical isolates with Illumina data.

The pDupI *pfgch1* promoter amplification was observed in field isolates from Kenya (KE01, 466bp region; chr. 12: 973,804–974,270; 3 copies) and Mali (378bp; chr. 12: 973,835–974,213; 2 copies), where differences in their length and number of copies suggest independent origin of these variations. Interpreting the Illumina pDupI amplifications, there appear to be at least three distinct events based on these breakpoints, two of them matching long-read sequencing denoted as pDupI_1 (KE01) and pDupI_3 (ML01), while in a third (pDupI_2) the start position overlaps with KE01 and the end with the ML01 strain (denoted KE01/ML01) (S1 Fig). All three types of pDupI amplifications contain a confirmed promoter (EPD promoter ID: CZT99398_1) and are frequent across African populations (n = 679, 32.1%; pDupI_1, n = 347, 16.4%; pDupI_2, n = 207, 9.8%; pDupI_3, n = 125, 5.9%) [24]. The pDupI_1 modification was the most prevalent amplification found in Southern (138/234, 59.0%) and East Africa (125/270, 46.3%), affecting Malawi (64.2%), Tanzania (46.8%) and Kenya (46.0%) (Table 2; S3 Table). For pDupI_2, the highest prevalence was observed in Southern (26.9%), East (18.5%) and Central Africa (11.9%), whilst pDupI_3 was found in West Africa (9.6%; Nigeria, 30.8%; Ghana, 18.4%; Ivory Coast, 10.9%; Mauritania, 10.9%).

In 7G8 (Brazil), D10 (Papua New Guinea) and K1 (Thailand) long-read sequences, we detected large copy number variants involving the *pfgch1* gene and its neighbouring loci (chr. 12 location: 7G8 946,264–978,039; D10 962,087–992,006; K1 961,455–980,630); however, no evidence of their existence was found in the clinical isolates using Illumina data (n = 4,134). Almost all of the reported breakpoints are located in non-coding regions and near to long repeats consisting of A and T nucleotides, confirming characteristics of copy number variant formation across the highly repetitive *P. falciparum* genome [25].

### Copy number variant analysis and frequency in global isolates

**Fig 2:**
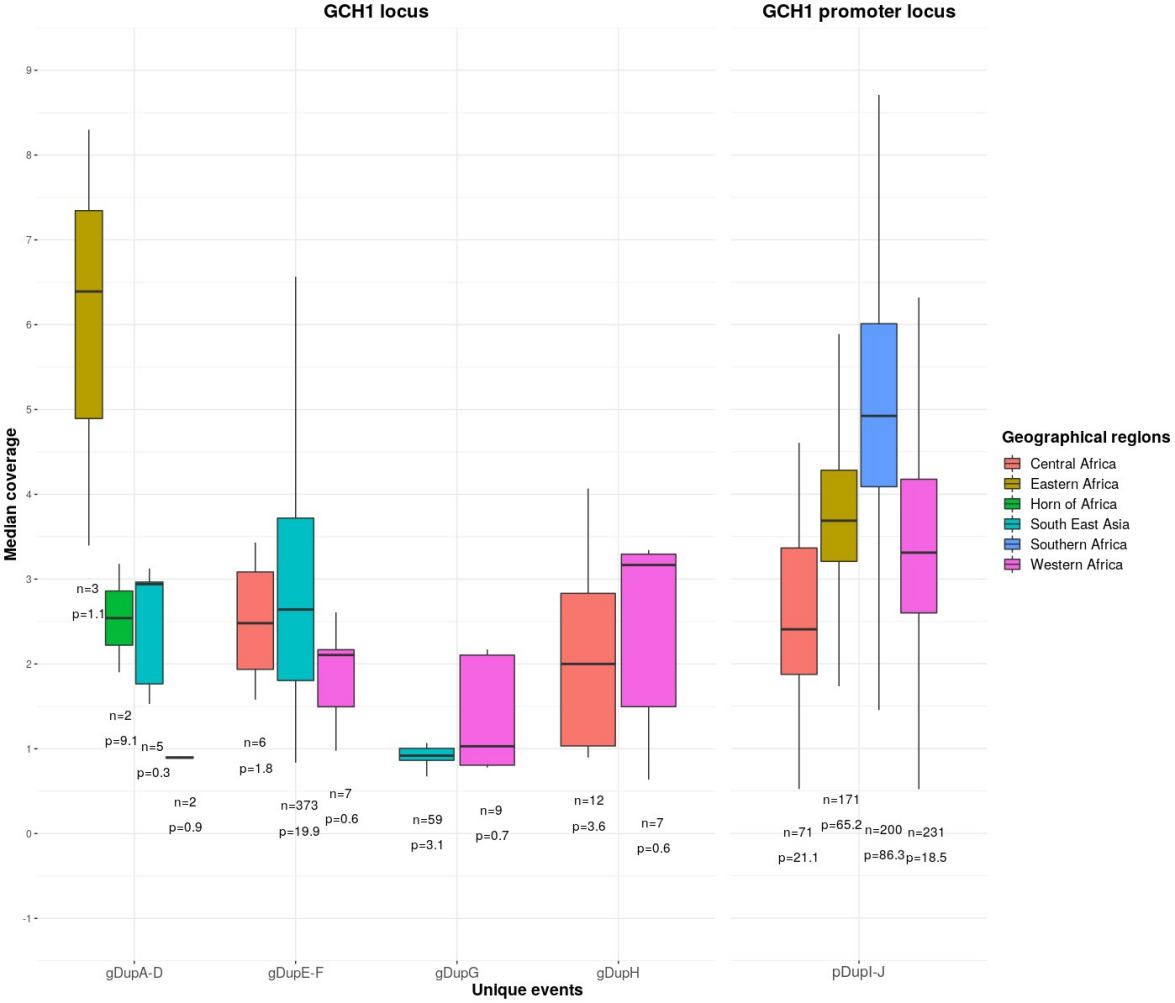
Copy numbers of the *pfgch1* amplifications. n—number of samples with amplification; p—percentage of samples with amplification.

Copy number variation in *pfgch1* has a potential role in sustaining SP resistance, and we estimated the number of copies directly using relative sequencing coverage compared to chromosome 12-wide levels of non-amplified isolates (Fig 2). For field isolates with gDupA, gDupB, and gDupC, we observed at least two copies, but in gDupD found in Madagascar there was only one copy. For gDupE and gDupF, there tended to be greater copies in Southeast Asia (median 2.66) than in West and Central Africa (median = 2.16) (Wilcoxon P = 0.03), which is consistent with the higher number of copies found in T996 (Thailand, 4 copies) compared to GB4 (Ghana, 3 copies) strains. The median number of copies of gDupG was approximately one. For gDupH events, there were at least two copies in Central Africa (median 2.00) and at least three copies for West African countries (median 3.17), consistent with the four copies of the NF54 strain. It is possible that pDupI and pDupJ have a common origin, but inference is difficult as there are only two pDupJ amplifications. There were differences in the the number of copies of the *pfgch1* promoter amplifications between regions in Africa (median: Southern 4.97, East 3.71, West 3.32, Central 2.41), consistent with differences in PacBio strains (copies: Kenya KE01 3, Mali ML01 2), and potentially linked to geographical differences in SP use.

### *Pfdhfr*/*pfdhps* mutations and *pfgch1* amplifications

It is known that the accumulation of *pfdhfr* and *pfdhps* mutations lead to greater resistance to the SP [26]. Moreover, the *pfgch1* amplification was found to facilitate further development of highly resistant *pfdhfr* parasites by improving their fitness and increasing resistance [27]. Multiple *pfdhfr*/*pfdhps* mutants and different *pfgch1* amplifications were frequently identified together, and there was a trend for those with amplifications to have a greater number of *pfdhfr*/*pfdhps* mutations in both Africa (Median number of *pfdhfr*/*pfdhps* mutations: No *pfgch1* amplification 4 vs. a *pfgch1* amplification 5; Wilcoxon P<0.0001) and Southeast Asia (No *pfgch1* amplification 5 vs. a *pfgch1* amplification 6; Wilcoxon P<0.0001) (S2 Fig). The most common amplifications in Southeast Asia (gDupE, gDupF and gDupG) were frequently (>80%) identified on a core quintuple (**IRN**IS-IS**GE**AA or **IRN**IS-IS**GN**AA) background, with at least six *pfdhfr*/*pfdhps* SNPs (**IRNL**S-IS**GEG**A 40.6%, **IRNL**S-I**AGE**AA 12.9%, **IRN**IS-I**AGE**AA 8.5%, **IRNL**S-IS**GNG**A 8.1%, **IRN**IS-IS**GEG**A (6.8%)) (S4 Table). The gDupH amplifications in West and Central Africa appear predominantly with the quadruple mutant (**IRN**IS-IS**G**KAA 50.0%), but a significant number (25%) were identified on the sextuple background (**IRN**IS-**VAG**K**GS** 15.0%, **IRN**IS-**VAG**KAA 10.0%). The promoter amplification pDupI ML01 amplification type was predominantly in West Africa, on two highly frequent *pfdhfr-pfdhps* mutant backgrounds (**IRN**IS-IS**G**KAA 31.2%, **IRN**IS-I**AG**KAA 25.6%). The remaining promoter amplifications (pDupI KE01 and KE01/ML01) were located primarily in Southern, East and Central Africa, and predominantly accompanied by a quintuple **IRN**IS-IS**GE**AA mutant (54.5%), as well as other frequent genotypes (**IRN**IS-IS**G**KAA 16.1%, **IRN**IS-IS**GEG**A 8.7%).

**Fig 3:**
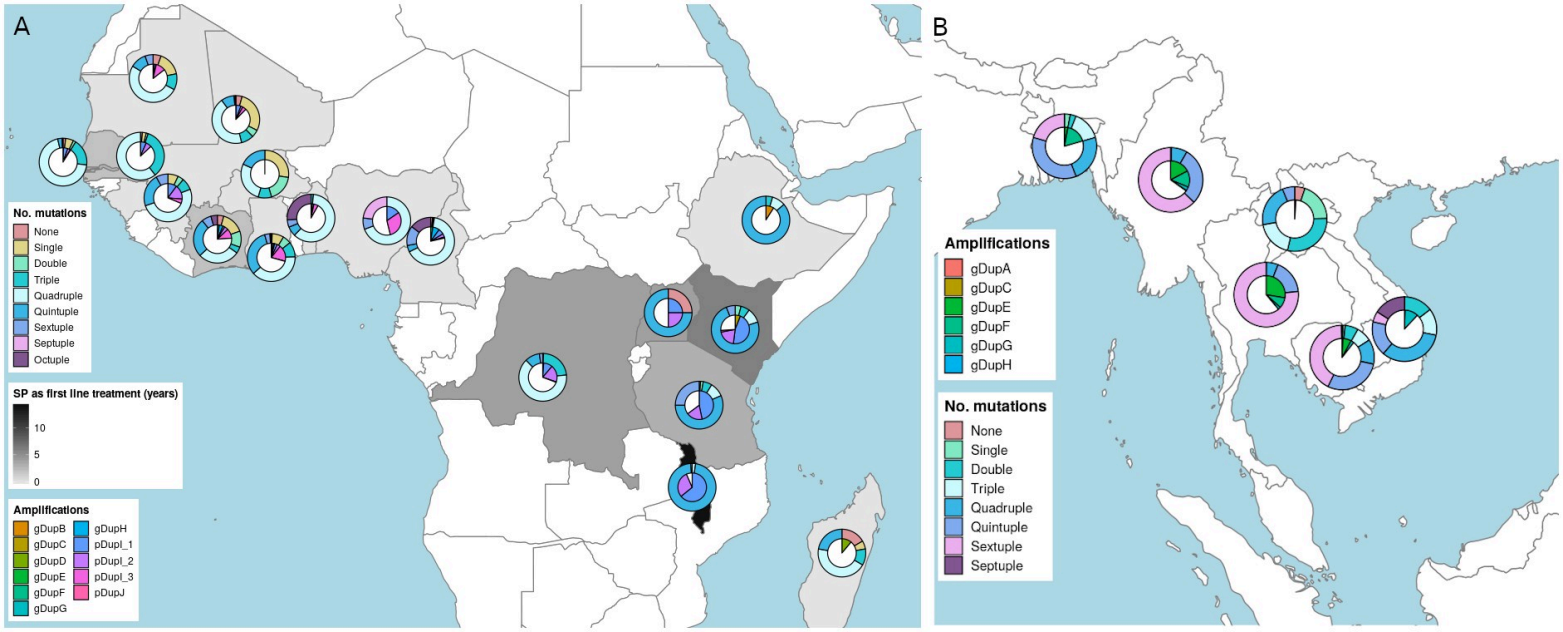
Maps with the distribution of the number of *pfdhfr/pfdhps* mutations (outer pie chart), *pfgch1* amplifications (inner pie chart) and sulfadoxine-pyrimethamine (SP) usage (shading of country)). A. Africa, B. South(east) Asia.

Despite the sampling period and frame of the data being “convenient” in nature rather than systematic, a time point analysis was possible using those countries with more than one year of sampling. This provided evidence of no change or increase in the frequency of *pfdhps*/*pfdhfr* mutants. For example, there was a higher frequency of quintuple and sextuple mutants in Kenya and Tanzania at later time-points (S3 Fig). There also appears to be an increasing number of *pfgch1* promoter amplifications in countries like Mali, Gambia and Kenya. In Kenya, from 2007 to 2014 the number of amplifications increased two-fold (S3 Fig), following the introduction of IPTp-SP in 1999 and six years of SP as first-line treatment (1998 to 2004). Almost 75% of Kenyan samples were reported with quintuple mutants and more than 65% contain *pfgch1* promoter amplifications (Fig 3). Similarly, for Tanzanian isolates, which were sourced between 2010 and 2013, 80% were found with quintuple or sextuple mutants and 65% reported promoter amplifications. In Tanzania, SP was used as a first-line treatment from 2001 to 2004 and IPTp-SP was introduced in 2001.

### Links to other drug resistance loci

The patterns of genotypic chloroquine (*pfcrt*, *pfmdr1*) and artemisinin (*pfkelch13*) drug resistance were explored in relation to the presence of SP related *pfdhfr*/*pfdhps* mutations and *pfgch1* amplifications (Table 3). Although, markers of chloroquine and artemisinin resistance are not in the folate pathway, they have been subject to directional selection, and an integrated analysis may reveal insights into the history of drug resistance in geographical regions. A high level of chloroquine resistance was observed across most of the geographical regions, except Southern Africa, where the Malawian population had almost no mutants and reversion to susceptible strains has been observed due to the early removal of chloroquine as a front-line antimalarial [1, 28] (Table 3). In West and Central Africa, those parasites with *pfgch1* gene amplifications (compared to promoter or no amplifications) had a lower prevalence of genotypic chloroquine resistance (Z-test P<2.2 × 10^−16^) (Table 3). The prevalence of genotypic chloroquine resistance in Southeast Asia is high (>80%) regardless of a *pfgch1* amplification, but a higher prevalence is reported in samples with gene amplifications (compared to none; Z-test P<2.2 × 10^−16^). As expected, artemisinin related mutations were only found in Southeast Asia, and a lower prevalence is associated with isolates with reported *pfgch1* amplifications (compared to none; Z-test P<8.3 × 10^−11^), but this analysis may be confounded by time of sampling. A temporal analysis revealed a decreasing frequency of chloroquine resistance related mutations (*pfcrt* K76T and I356T; *pfmdr1* N86Y) in Eastern Africa (Tanzania: 2010 71.9%, 2013 30.1%; Kenya: 2007 80%, 2014 7.1%) (S3 Fig). There were no strong signals of *pfcrt* resistance reversion in any of the Southeast Asian populations, but the data only spanned 2007 to 2014, and chloroquine is still accessible and used due to the continued presence of *P. vivax* [29].

**Table 3:** Distribution of the *pfgch1* amplifications (Dup).

Region	Dup *	n	No. *pfdhfr*/*pfdhps* mutations ** Mean/Median [IQR]	CQ Resistance *** (%)	ARS Resistance **** (%)
West Africa	None	999	3.6/4 [3-4]	50.7	-
	Prom	232	4.0/4 [4-5]	50.0	-
	Gene	23	4.0/4 [3-4.5]	26.1	-
Central Africa	None	248	4.3/4 [4-4]	66.5	-
	Prom	71	4.2/4 [4-4]	67.6	-
	Gene	18	4.8/4 [4-5]	72.2	-
East Africa	None	94	4.5/5 [4-5]	34.0	-
	Prom	176	5.1/5 [5-6]	35.8	-
Southern Africa	None	32	4/5 [4-5]	28.1	-
	Prom	202	5.0/5 [5-5]	1.0	-
Horn of Africa	None	22	4.8/5 [5-5]	90.9	-
South(east) Asia	None	1453	5.9/6 [5-7]	83.7	35.7
	Gene	437	6.6/7 [6-7]	91.8	22.2
Oceania	None	95	3.6/3 [3-5]	97.9	-
South America	None	32	1.9/1 [1-2.25]	100	-

CQ chloroquine; ARS artemisinin; IQR interquartile range;

* Promoter pDupI-J; Gene gDupA-H;

** based on *pfdhfr* (N51I, C59R, S108N, I164L and S306F) and *pfdhps* (I431V, S436A, A437G, K540E/K540N, A581G and S613S);

*** based on *pfcrt* K76T and other mutations (C72S, M74I, N75D/E, A144F, L148I, I194T, A220S, Q271E, A326D/S, I356L/T and R371I) or *pfmdr1* N86Y;

**** based on *pfkelch13* (F446I, Y493H, P574L, R539T, and C580Y)

## Discussion

Monitoring of the polymorphisms in the *P. falciparum* genome associated with antimalarial drug resistance can be used to control and prevent malaria, support elimination strategies, and guide treatment choices. Surveillance is crucial for drugs like SP, which was replaced as a front-line treatment, and is now used prophylactically in pregnant women and children. Increasing numbers of *pfdhfr* and *pfdhps* resistance mutants due to selection by drug pressure have been observed, and increased copy number of *pfgch1*, which encodes the first and the rate-limiting enzyme of *de novo* folate biosynthesis, has been linked to SP resistance in Southeast Asia, particularly Thailand [8]. Adaptations in *pfgch1* gene are thought to compensate for the lower fitness in parasites with three or more mutations in *pfdhfr* or increase resistant in parasites with fewer polymorphisms [27]. The function of the promoter duplication remains unknown, however, it is plausible that it performs a similar role as the gene amplification. Moreover, multiple copies of *pfgch1* have been shown to have a direct association with point mutations in *pfdhfr* (I164L) or *pfdhps* (K540E) in Southeast Asia [9], which is confirmed in our work. Similarly, in Malawi and other countries in Africa which have had prolonged SP use, a *pfgch1* promoter duplication has been identified in parasites with *pfdhfr*/*pfdhps* mutations, distinct from the whole gene amplification found in Southeast Asia.

Our analysis in 4,134 *P. falciparum* across 29 malaria endemic countries reveals a more fine-scaled picture of *pfgch1* gene and promoter amplifications, reporting 12 different structural changes occurring in African or Asian populations. Using 18 strains with long read sequence data allowed us to robustly determine the breakpoints, including in regions of long mono-nucleotide (A or T) or AT/TA di-nucleotide repeats. The prevalence of amplifications was correlated with geography, with gene- and promoter-based types dominant in Southeast Asia and Africa, respectively. The divergence between continents and multiple types indicate an independent emergence of structural changes. Furthermore, the location of breakpoints in areas of mono- or di-nucleotide repeats, encourage even more distinctive variations due to an AT-rich genome of *P. falciparum*. Nonetheless, there were minor exceptions, including in Cameroon, DRC, Kenya and Ghana [10], where both promoter and gene amplifications were present. Further, the extent of amplification copy number appears to be linked to geography. For example, the Ghanaian (GB4) and Thailand (T996) strains had identical breakpoints, but the latter has one additional copy. Similarly, for the promoter amplification DupI, isolates from Southern and East African countries tended to have higher estimated copy number compared to the western and central regions of the continent. This regional difference is in part due to local evolution of strains exposed to SP (and other anti-folate drugs) over a longer period, resulting from differences in antimalarial drug implementation policy between countries.

Higher numbers of *pfdhfr*/*pfdhps* mutations were correlated with the presence of *pfgch1* amplifications in both Africa and Asia. Whilst, the relatively low numbers of *pfdhfr*/*pfdhps* mutations in Papua New Guinea and South America were accompanied by the absence of *pfgch1* amplifications. The promoter amplification (DupI) and quintuple *pfdhfr*/*pfdhps* genotype (**IRN**IS-IS**GE**AA) were linked in African populations, whilst the septuple (**IRNL**S-IS**GEG**A) mutant was linked with gene amplifications (DupE and DupF) in Asia. The prevalence of *pfgch1* gene and promoter amplifications and occurrence of *pfdhfr*/*pfdhps* mutants in Africa broadly overlaps with the duration and degree of SP usage as a first-line treatment. Malawi abandoned chloroquine early and has had the longest SP exposure with >10 years as a first-line treatment, and almost all isolates contained *pfgch1* promoter amplifications, *pfdhfr*/*pfdhps* quintuple mutants and *pfcrt* wild-type alleles. Whereas, Kenya and Tanzania introduced SP five or more years after Malawi, and ∼65% isolates had promoter amplifications and a low prevalence of *pfcrt* resistance alleles (<40%). Further, across the seven years of data from Kenya, there was an increase in both the prevalence of *pfgch1* promoter amplifications and *pfdhfr*/*pfdhps* quintuple mutants. In Southeast Asia the near fixation of chloroquine resistance alleles and higher levels of SP related *pfdhfr*/*pfdhps* mutations, were in tandem with 20% *pfgch1* amplification frequency, particularly in Thailand and Myanmar. Interestingly, in Southeast Asia there was an allelic association between *pfcrt* I356T and *pfdhfr* I164L, most probably due to the high historical usage of both antimalarial drugs.

Overall, whilst there may be some limitations with the convenience nature of the *P. falciparum* sampling, the trend towards longer SP exposure and an increase in drug resistance polymorphisms makes it essential to monitor structural changes in the *pfgch1* locus and the number of *pfdhfr*/*pfdhps* mutations as well as correlations between them. *Pfgch1* gene expression may modify SP sensitivity [30], and further analysis studying the expression of different *pfgch1* amplifications with relation to *pfdhfr*/*pfdhps* could provide additional insights into SP resistance. In lieu of further whole genome sequencing, drug resistance assays, and allelic manipulation of *P. falciparum* to understand functional mechanisms, our work has characterised new forms of *pfgch1* amplification, which can be used as the basis of enhanced surveillance for SP efficacy.

## Conclusion

The SP combination is still the only antimalarial drug treatment recommended by WHO for intermittent preventive treatment in vulnerable populations, because of its safety in pregnant women and infants and its long action. The selection of *P. falciparum* with *pfgch1* amplifications may enhance the fitness of parasites with *pfdhfr* and *pfdhps* substitutions, intensifying the persistence of SP resistance, and potentially threatening the efficacy of this regimen for prevention of malaria in vulnerable groups. Our work has revealed new forms of *pfgch1* amplification, which can be used for surveillance activities.

## Supporting information

S1 Fig Histogram of the start and end positions for the three types of *pfgch1* promoter amplifications identified (KE01, ML01, KE01/ML01). (PDF)

S2 Fig The proportion of *pfgch1* amplifications (none—red; gene—green; promoter—blue) by the number *pfdhfr* and *pfdhps* mutations*. A. Africa, B. South(east) Asia.
(PDF)

S3 Fig Number of *pfdhfr*/*pfdhps* mutations and *pfgch1* promoter amplifications over time for five African countries with more than one year of sampling. (PDF)

S1 Table The geographical distribution of common *pfdhfr* and*pfdhps* mutations in 4,134*P. falciparum* isolates (%). (PDF)

S2 Table Frequency (%) of the *pfdhfr*/*pfdhps* genotypes by country. (PDF)

S3 Table Frequency (%) of the *pfgch1* amplifications (DupA-J) by country. (PDF)

S4 Table The main *pfgch1* amplifications with the most frequent *pfdhfr*/*pfdhps* genotypes in Africa and Asia (%). (PDF)

## References

[1] Ravenhall M, Benavente ED, Mipando M, Jensen ATR, Sutherland CJ, Roper C, et al Characterizing the impact of sustained sulfadoxine/pyrimethamine use upon the Plasmodium falciparum population in Malawi. Malaria Journal. 2016;15(1):575 10.1186/s12936-016-1634-6 27899115 PMC5129638

[2] Ravenhall M, Benavente ED, Sutherland CJ, Baker DA, Campino S, Clark TG. An analysis of large structural variation in global Plasmodium falciparum isolates identifies a novel duplication of the chloroquine resistance associated gene. Scientific Reports. 2019;9(1):8287 10.1038/s41598-019-44599-0 31164664 PMC6547842

[3] Antimalarial drug resistance: linking Plasmodium falciparum parasite biology to the clinic. Parasite Biology To the Clinic. Nature Medicine. 2017;23(8):917–928. 10.1038/nm.4381 28777791 PMC5747363

[4] Sutherland CJ, Fifer H, Pearce RJ, Bin Reza F, Nicholas M, Haustein T, et al Novel pfdhps haplotypes among imported cases of Plasmodium falciparum malaria in the United Kingdom. Antimicrobial Agents and Chemotherapy. 2009;53(8):3405–3410. 10.1128/AAC.00024-09 19433569 PMC2715629

[5] Oguike MC, Falade CO, Shu E, Enato IG, Watila I, Baba ES, et al Molecular determinants of sulfadoxine-pyrimethamine resistance in Plasmodium falciparum in Nigeria and the regional emergence of dhps 431V. International Journal for Parasitology: Drugs and Drug Resistance.2016;6(3):220–229. 10.1016/j.ijpddr.2016.08.004 27821281 PMC5094156

[6] van Eijk AM, Larsen DA, Kayentao K, Koshy G, Slaughter DEC, Roper C, et al Effect of Plasmodium falciparum sulfadoxine-pyrimethamine resistance on the effectiveness of intermittent preventive therapy for malaria in pregnancy in Africa: a systematic review and meta-analysis. The Lancet Infectious Diseases. 2019;19(5):546–556 30922818 10.1016/S1473-3099(18)30732-1

[7] Juma DW, Muiruri P, Yuhas K, John-Stewart G, Ottichilo R, Waitumbi J, et al The prevalence and antifolate drug resistance profiles of Plasmodium falciparum in study participants randomized to discontinue or continue cotrimoxazole prophylaxis. PLoS Neglected Tropical Diseases. 2018;13(3):1–12 10.1371/journal.pntd.0007223 PMC6445470 30897090

[8] Nair S, Miller B, Barends M, Jaidee A, Patel J, Mayxay M, et al Adaptive copy number evolution in malaria parasites. PLoS Genetics. 2008;4(10). 10.1371/journal.pgen.1000243 18974876 PMC2570623

[9] Sugaram R, Suwannasin K, Kunasol C, Mathema VB, Day NPJ, Sudathip P, et al Molecular characterization of Plasmodium falciparum antifolate resistance markers in Thailand between 2008 and 2016. Malaria Journal. 2020;19(1):1–10. 10.1186/s12936-020-03176-x 32127009 PMC7055081

[10] Osei M, Ansah F, Matrevi SA, Asante KP, Awandare GA, Quashie NB, et al Amplification of GTP-cyclohydrolase 1 gene in plasmodium falciparum isolates with the quadruple mutant of dihydrofolate reductase and dihydropteroate synthase genes in Ghana. PLoS ONE. 2018;13(9):1–13. 10.1371/journal.pone.0204871 30265714 PMC6162080

[11] MalariaGEN Plasmodium falciparum Community Project. An open dataset of Plasmodium falciparum genome variation in 7,000 worldwide samples. bioRxiv. 2019 10.12688/wellcomeopenres.16168.1 PMC8008441 33824913

[12] Assefa SA, Preston MD, Campino S, Ocholla H, Sutherland CJ, Clark TG. EstMOI: Estimating multiplicity of infection using parasite deep sequencing data. Bioinformatics. 2014;30(9):1292–1294. 24443379 10.1093/bioinformatics/btu005 PMC3998131

[13] Benavente ED, Oresegun DR, de Sessions PF, Walker EM, Roper C, Dombrowski JG, et al Global genetic diversity of var2csa in Plasmodium falciparum with implications for malaria in pregnancy and vaccine development. Scientific Reports. 2018;8(1):1–8. 10.1038/s41598-018-33767-3 30337594 PMC6193930

[14] Amambua-Ngwa A, Amenga-Etego L, Kamau E, Amato R, Ghansah A, Golassa L, et al Major subpopulations of Plasmodium falciparum in sub-Saharan Africa. Science. 2019;365(6455):813–816. 10.1126/science.aav5427 31439796

[15] Trager W, Jensen J. Human malaria parasites in continuous culture. Science. 1976;193(4254):673–675. 781840 10.1126/science.781840

[16] Otto TD, Böhme U, Sanders M, Reid A, Bruske EI, Duffy CW, et al Long read assemblies of geographically dispersed Plasmodium falciparum isolates reveal highly structured subtelomeres [version 1; referees: 3 approved]. Wellcome Open Research. 2018;3(May):1–24. 10.12688/wellcomeopenres.14571.1 29862326 PMC5964635

[17] Darling ACE, Mau B, Blattner FR, Perna NT. Implicitfunction.Pdf. Genome Research. 2004;14:1394–1403. 10.1101/gr.2289704 15231754 PMC442156

[18] Ecker A, Lehane AM, Clain J, Fidock DA. PfCRT and its role in antimalarial drug resistance. Trends in Parasitology. 2012;28(11):504–514. 23020971 10.1016/j.pt.2012.08.002 PMC3478492

[19] Atroosh WM, Al-Mekhlafi HM, Mahdy MAK, Surin J. The detection of pfcrt and pfmdr1 point mutations as molecular markers of chloroquine drug resistance, Pahang, Malaysia. Malaria Journal. 2012;11(1):1. 22853645 10.1186/1475-2875-11-251 PMC3493286

[20] Andriantsoanirina V, Ratsimbasoa A, Bouchier C, Tichit M, Jahevitra M, Rabearimanana S, et al Chloroquine clinical failures in P. falciparum malaria are associated with mutant Pfmdr-1, not Pfcrt in madagascar. PLoS ONE. 2010;5(10). 10.1371/journal.pone.0013281 20967251 PMC2954150

[21] He Y, Campino S, Benavente ED, Warhurst DC, Beshir KB, Lubis I, et al Artemisinin resistance-associated markers in Plasmodium falciparum parasites from the China-Myanmar border: Predicted structural stability of K13 propeller variants detected in a low-prevalence area. PLoS ONE. 2019;14(3):1–13. 10.1371/journal.pone.0213686 PMC6422288 30883571

[22] Mandoko PN, Rouvier F, Kakina LM, Mbongi DM, Latour C, Likwela JL, et al Prevalence of plasmodium falciparum parasites resistant to sulfadoxine/pyrimethamine in the democratic republic of the congo: Emergence of highly resistant PfdHFR/PfdHps alleles. Journal of Antimicrobial Chemotherapy. 2018;73(10):2704–2715. 10.1093/jac/dky258 30053021

[23] Jiang T, Chen J, Fu H, Wu K, Yao Y, Eyi JUM, et al High prevalence of Pfdhfr-Pfdhps quadruple mutations associated with sulfadoxine-pyrimethamine resistance in Plasmodium falciparum isolates from Bioko Island, Equatorial Guinea. Malaria Journal. 2019;18(1):1–8. 10.1186/s12936-019-2734-x 30914041 PMC6434785

[24] Adjalley SH, Chabbert CD, Klaus B, Pelechano V, Steinmetz LM. Landscape and Dynamics of Transcription Initiation in the Malaria Parasite Plasmodium falciparum. Cell Reports. 2016;14(10):2463–2475. 10.1016/j.celrep.2016.02.025 26947071 PMC4806524

[25] Huckaby AC, Granum CS, Carey MA, Szlachta K, Al-Barghouthi B, Wang YH, et al Complex DNA structures trigger copy number variation across the Plasmodium falciparum genome. Nucleic Acids Research. 2019;47(4):1615–1627. 10.1093/nar/gky1268 30576466 PMC6393310

[26] Mita T, Ohashi J, Venkatesan M, Marma ASP, Nakamura M, Plowe CV, et al Ordered accumulation of mutations conferring resistance to sulfadoxine-pyrimethamine in the plasmodium falciparum parasite. Journal of Infectious Diseases. 2014;209(1):130–139. 23922363 10.1093/infdis/jit415

[27] Kümpornsin K, Modchang C, Heinberg A, Ekland EH, Jirawatcharadech P, Chobson P, et al Origin of robustness in generating drug-resistant malaria parasites. Molecular Biology and Evolution. 2014;31(7):1649–1660. 10.1093/molbev/msu140 24739308 PMC4069624

[28] Fong P, Boss D, Yap T, Tutt A, Wu P, Mergui-Roelvink M. New England Journal CREST. Science. 2010; p. 609–619.20688985

[29] Commons RJ, Simpson JA, Thriemer K, Humphreys GS, Abreha T, Alemu SG, et al The effect of chloroquine dose and primaquine on Plasmodium vivax recurrence: a WorldWide Antimalarial Resistance Network systematic review and individual patient pooled meta-analysis. The Lancet Infectious Diseases. 2018;18(9):1025–1034. 10.1016/S1473-3099(18)30348-7 30033231 PMC6105624

[30] Heinberg A, Siu E, Stern C, Lawrence EA, Ferdig MT, Deitsch KW, et al Direct evidence for the adaptive role of copy number variation on antifolate susceptibility in Plasmodium falciparum. Mol Microbiol. 2013;88(4). 10.1111/mmi.12162 23347134 PMC3654098

