# Supplementary material for: Genetic diversity of the Plasmodium falciparum GTP-cyclohydrolase 1, dihydrofolate reductase and dihydropteroate synthetase genes reveals new insights into sulfadoxine-pyrimethamine antimalarial drug resistance
Source: PLoS Genet. 2020 Dec 31;16(12):e1009268. doi: 10.1371/journal.pgen.1009268 (PMC7774857; doi:10.1371/journal.pgen.1009268)

S1 Fig. Histogram of the start and end positions for the three types of *pfgch1* promoter amplifications identified (KE01, ML01, KE01/ML01).

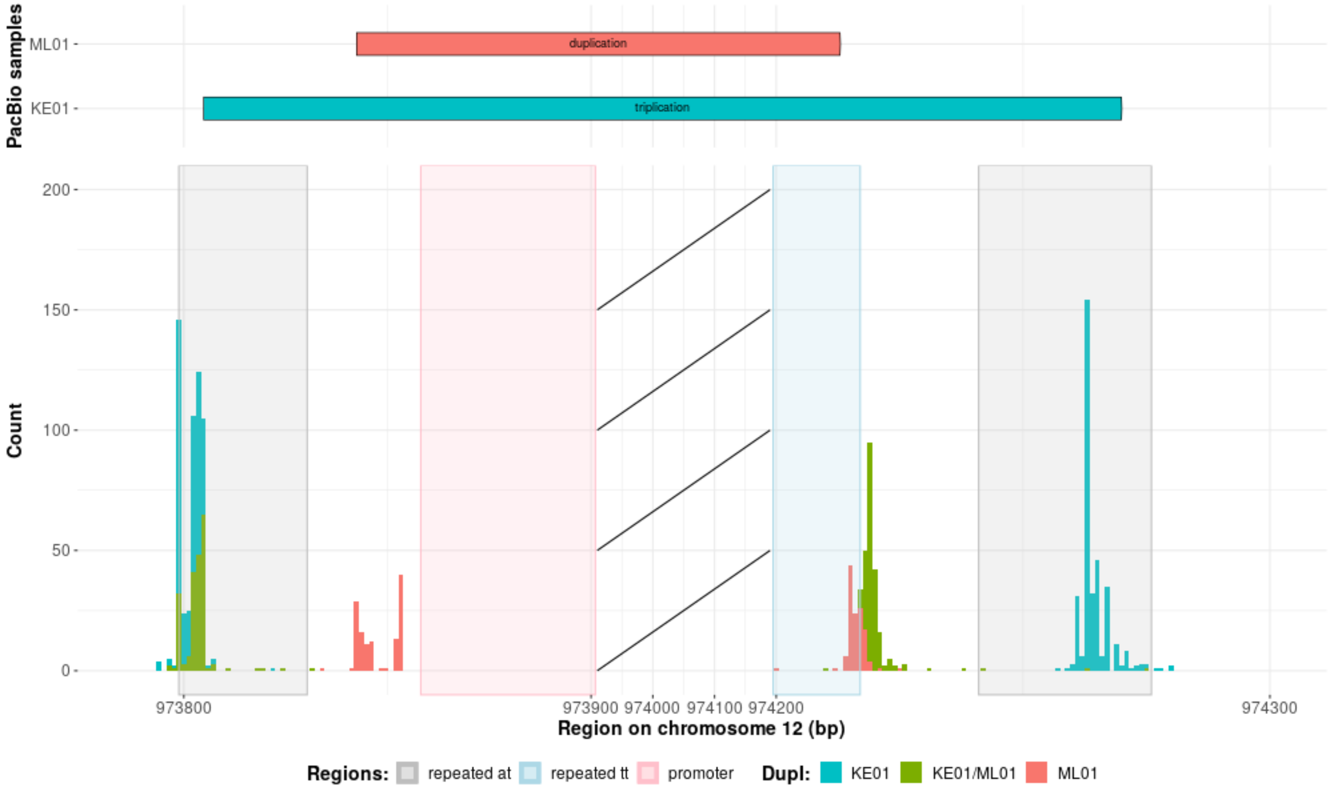

Supplement: S1 Fig — (PDF) [file pgen.1009268.s001.pdf]
