# Supplementary material for: Genetic diversity of the Plasmodium falciparum GTP-cyclohydrolase 1, dihydrofolate reductase and dihydropteroate synthetase genes reveals new insights into sulfadoxine-pyrimethamine antimalarial drug resistance
Source: PLoS Genet. 2020 Dec 31;16(12):e1009268. doi: 10.1371/journal.pgen.1009268 (PMC7774857; doi:10.1371/journal.pgen.1009268)
Supplement: S2 Fig — A. Africa, B. South(east) Asia. (PDF) [file pgen.1009268.s002.pdf]

S2 Fig. The proportion of *pfgch1* amplifications by the number *pfdhfr* and *pfdhps* mutations*. A. Africa, B. South(east) Asia

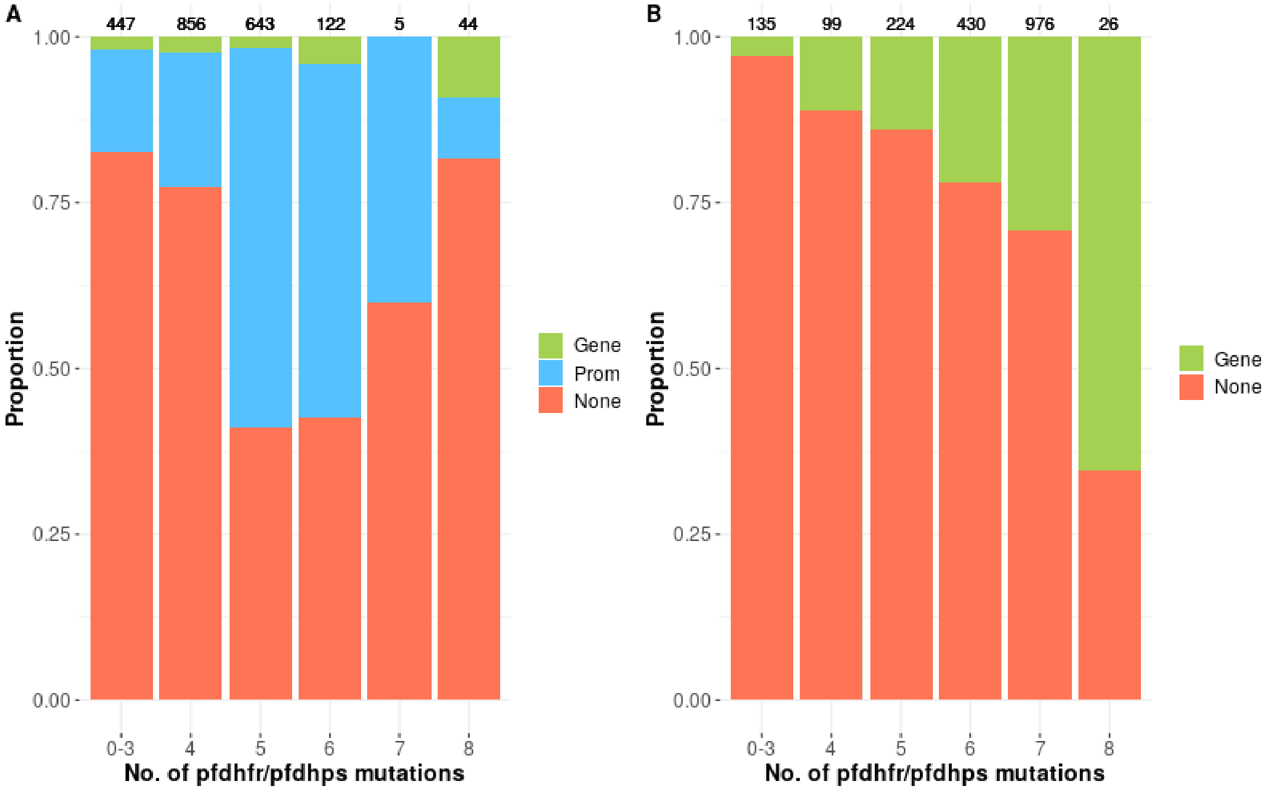

* based on *pfdhfr* (N51I, C59R, S108N and I164L) and *pfdhps* (S436A, A437G, K540E/K540N, A581G and S613A)
