# Supplementary material for: Genetic diversity of the Plasmodium falciparum GTP-cyclohydrolase 1, dihydrofolate reductase and dihydropteroate synthetase genes reveals new insights into sulfadoxine-pyrimethamine antimalarial drug resistance
Source: PLoS Genet. 2020 Dec 31;16(12):e1009268. doi: 10.1371/journal.pgen.1009268 (PMC7774857; doi:10.1371/journal.pgen.1009268)
Supplement: S3 Fig — (PDF) [file pgen.1009268.s003.pdf]

S3 Fig. Number of *pfdhfr*/*pfdhps* mutations and *pfgch1* promoter amplifications over time for five African countries with more than one year of sampling.

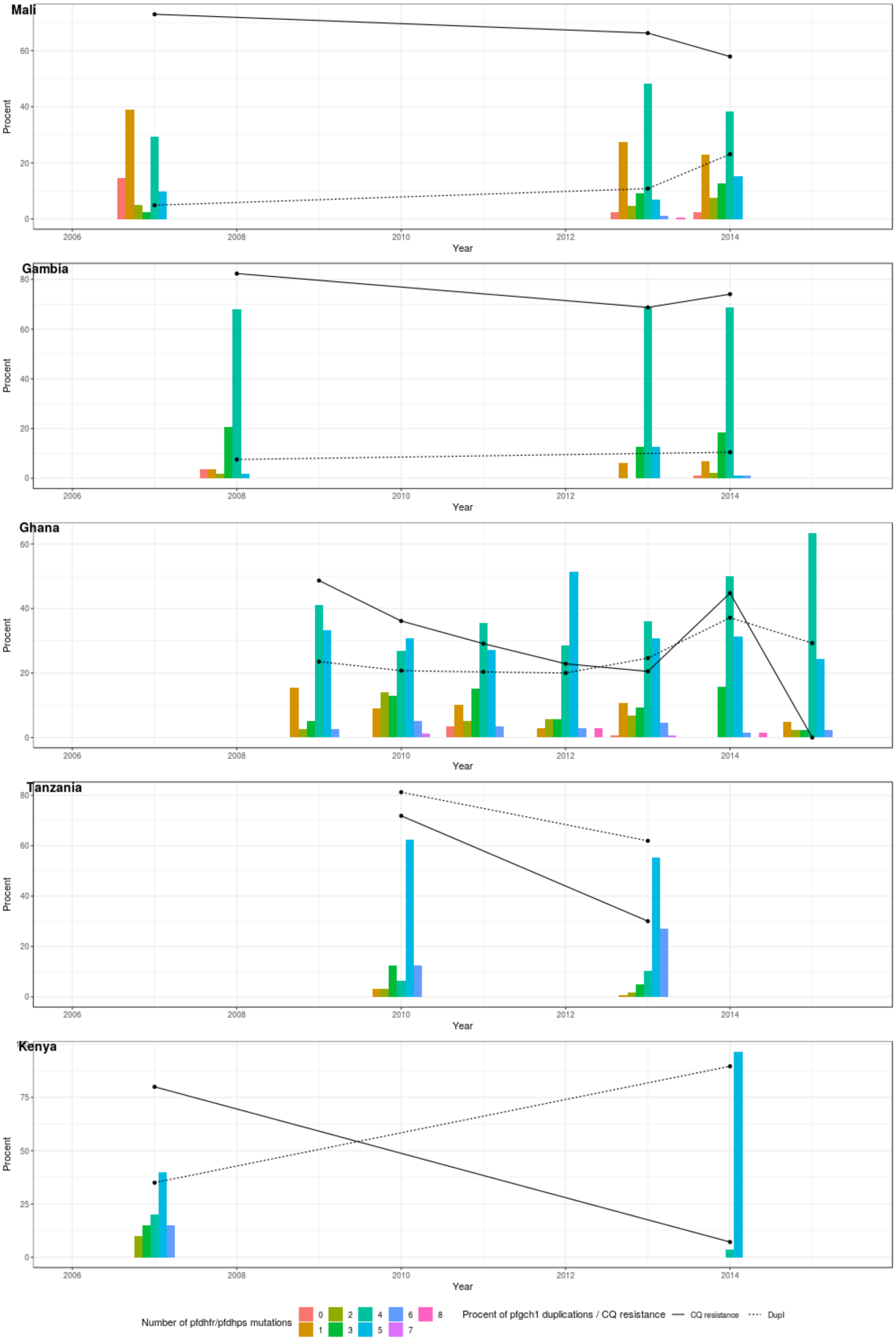
Mali
Gambia
Ghana
Tanzania
Kenya
Procent
Year
2006
2008
2010
2012
2014
0
20
40
60
80
25
50
75
Number of pfdhfr/pfdhps mutations
0
1
2
3
4
5
6
7
8
Procent of pfgch1 duplications / CQ resistance
CQ resistance
Dupl
