# Supplementary material for: Genetic diversity of the Plasmodium falciparum GTP-cyclohydrolase 1, dihydrofolate reductase and dihydropteroate synthetase genes reveals new insights into sulfadoxine-pyrimethamine antimalarial drug resistance
Source: PLoS Genet. 2020 Dec 31;16(12):e1009268. doi: 10.1371/journal.pgen.1009268 (PMC7774857; doi:10.1371/journal.pgen.1009268)
Supplement: S1 Table — (PDF) [file pgen.1009268.s004.pdf]

S1 Table. The geographical distribution of common *pfdhfr* and *pfdhps* mutations in 4,134 *P. falciparum* isolates (%).

Region	n	*pfdhfr*					*pfdhps*							
		N51I	C59R	S108N	I164L	S306F	I431V	S436A	S436F	A437G	K540E	K540N	A581G	A613S
West Africa	1254	**74.1**	**80.1**	**81.9**	-	-	2.1	**50.3**	0.8	**68.8**	1.0	-	1.9	8.0
Central Africa	337	**98.2**	**86.4**	**99.4**	-	-	11.0	22.6	-	**93.5**	4.7	-	8.9	8.9
East Africa	270	**95.2**	**90.4**	**98.5**	1.5	-	-	3.7	1.5	**88.5**	**87.4**	-	21.1	0.4
Southern Africa	234	**96.6**	**97.9**	**98.3**	-	-	-	0.4	-	**97.9**	**92.3**	-	2.1	-
Horn of Africa	22	**100**	**90.9**	**100**	-	-	-	-	-	**95.5**	**95.5**	-	-	-
South(east) Asia	1890	**89.6**	**99.6**	**99.6**	**60.2**	-	-	29.7	1.7	**92.9**	**61.9**	18.8	**52.3**	1.5
Oceania	95	-	**100**	**100**	-	**84.2**	-	-	-	42.1	32.6	-	-	-
South America	32	28.1	-	**93.8**	6.3	-	-	-	-	28.1	9.4	-	21.9	-

Frequencies greater than 50% are bolded
