# Supplementary material for: Genetic diversity of the Plasmodium falciparum GTP-cyclohydrolase 1, dihydrofolate reductase and dihydropteroate synthetase genes reveals new insights into sulfadoxine-pyrimethamine antimalarial drug resistance
Source: PLoS Genet. 2020 Dec 31;16(12):e1009268. doi: 10.1371/journal.pgen.1009268 (PMC7774857; doi:10.1371/journal.pgen.1009268)
Supplement: S2 Table — (PDF) [file pgen.1009268.s005.pdf]

S2 Table. Frequency (%) of *pfdhfr*/*pfdhps* genotypes by country.

Genotypes *	Mauritania	Mali	Senegal	Gambia	Guinea	Burkina Faso	Ivory Coast	Ghana	Benin	Nigeria	Cameroon	DRC	Uganda	Kenya	Tanzania	Malawi	Madagascar	Ethiopia
NCSIS-ISAKAA	5.5	4.3	1.3	1.8	-	-	4.3	0.6	-	-	1.3	-	**25.0**	-	-	-	**16.7**	-
NCSIS-IS**G**KAA	3.6	**10.5**	-	3.0	3.0	-	6.5	6.2	-	-	-	-	-	-	-	-	5.6	-
NCSIS-I**A**AKAA	**12.7**	**17.8**	2.6	2.4	4.0	**18.2**	8.7	1.5	-	-	-	-	-	-	0.9	-	-	-
NCSIS-I**AG**KAA	-	3.9	1.3	0.6	3.0	**18.2**	6.5	4.9	-	-	-	-	-	-	-	-	-	-
NC**N**IS-ISAKAA	-	-	-	-	-	-	-	-	-	-	-	-	-	-	-	-	-	-
N**RN**IS-ISAKAA	-	0.8	-	-	-	-	-	-	-	-	-	-	-	-	0.5	-	-	-
N**RN**IS-IS**G**KAA	1.8	1.2	-	1.2	3.0	-	-	6.2	1.6	-	-	0.6	-	-	-	-	-	-
N**RN**IS-IS**GE**AA	-	-	-	-	-	-	-	0.2	-	-	-	-	-	2.0	2.3	1.9	-	-
N**RN**IS-I**A**AKAA	1.8	1.9	-	-	1.0	-	-	1.9	-	-	-	-	-	-	0.5	-	-	-
N**RN**IS-I**AG**KAA	-	-	-	-	1.0	-	6.5	4.3	-	-	-	0.6	-	-	-	-	-	-
N**RN**IS-I**AGE**AA	-	-	-	-	-	-	-	-	-	-	-	-	-	-	-	-	-	-
N**RN**I**F**-ISAKAA	-	-	-	-	-	-	-	-	-	-	-	-	-	-	-	-	-	-
N**RN**I**F**-IS**G**KAA	-	-	-	-	-	-	-	-	-	-	-	-	-	-	-	-	-	-
N**RN**I**F**-IS**GE**AA	-	-	-	-	-	-	-	-	-	-	-	-	-	-	-	-	-	-
N**RNL**S-IS**GEG**A	-	-	-	-	-	-	-	-	-	-	-	-	-	-	-	-	-	-
N**RNL**S-I**AGE**AA	-	-	-	-	-	-	-	-	-	-	-	-	-	-	-	-	-	-
**I**CN**I**S-ISAKAA	-	-	-	-	-	-	-	-	-	-	-	-	-	4.0	0.9	-	-	-
**I**C**N**IS-IS**G**KAA	1.8	-	-	1.8	-	-	-	1.1	-	-	-	**18.4**	-	-	-	-	-	-
**I**C**N**IS-IS**GE**AA	-	-	-	-	-	-	-	-	-	-	-	2.2	-	8.0	4.6	0.5	-	9.1
**I**C**N**IS-IS**GEG**A	-	-	-	-	-	-	-	-	-	-	-	1.1	-	-	0.9	-	-	-
**IRN**IS-ISAKAA	3.6	4.7	**32.9**	**14.4**	4.0	9.1	2.2	0.4	-	-	0.6	3.4	-	6.0	5.1	-	**11.1**	4.5
**IRN**IS-IS**G**KAA	**20.0**	**16.3**	**34.2**	**55.1**	**36.4**	9.1	**19.6**	**24.8**	**57.4**	**69.2**	**59.5**	**59.2**	-	-	-	-	**44.4**	-
**IRN**IS-IS**G**K**G**A	-	-	-	-	-	-	-	-	-	-	-	-	-	-	-	-	**22.2**	-
**IRN**IS-IS**GNG**A	-	-	-	-	-	-	-	-	-	-	-	-	-	-	-	-	-	-
**IRN**IS-IS**GE**AA	3.6	-	-	0.6	4.0	-	-	0.9	-	-	-	3.4	**75.0**	**72.0**	**54.6**	**96.7**	0.0	**86.4**
**IRN**IS-IS**GEG**A	-	-	-	-	-	-	-	-	-	-	-	2.2	-	-	**25.0**	0.5	-	-
**IRN**IS-I**A**AKAA	**30.9**	**27.5**	**25.0**	**14.4**	**12.1**	9.1	2.2	7.9	1.6	-	5.7	1.1	-	-	2.8	-	-	-
**IRN**IS-I**AG**KAA	7.3	8.5	-	2.4	**16.2**	**18.2**	**23.9**	**28.8**	6.6	-	4.4	5.6	-	-	-	-	-	-
**IRN**IS-I**AG**KA**S**	5.5	0.8	-	0.6	8.1	0.0	6.5	3.6	4.9	7.7	3.8	-	-	-	-	-	-	-
**IRN**IS-I**AGE**AA	-	-	-	-	-	-	-	-	-	-	0.0	-	-	-	-	0.5	-	-
**IRN**IS-**VAG**KAA	-	-	-	-	-	-	-	-	-	-	8.9	-	-	-	-	-	-	-
**IRN**IS-**VAG**K**GS**	-	0.4	-	-	-	-	4.3	0.4	**26.2**	-	**14.6**	-	-	-	-	-	-	-
**IRNL**S-IS**GNG**A	-	-	-	-	-	-	-	-	-	-	-	-	-	-	-	-	-	-
**IRNL**S-IS**GE**AA	-	-	-	-	-	-	-	-	-	-	-	-	-	-	-	-	-	-
**IRNL**S-IS**GEG**A	-	-	-	-	-	-	-	-	-	-	-	-	-	-	-	-	-	-
**IRNL**S-I**FG**KAA	-	-	-	-	-	-	-	-	-	-	-	-	-	6.0	-	-	-	-
**IRNL**S-I**FGE**A**S**	-	-	-	-	-	-	-	-	-	-	-	-	-	-	-	-	-	-
**IRNL**S-I**AGE**AA	-	-	-	-	-	-	-	-	-	-	-	-	-	-	-	-	-	-

WT = wild-type; DRC = Democratic Republic of Congo; PNG = Papua New Guinea; * based on *pfdhfr* (N51I, C59R, S108N and I164L) and *pfdhps* (S436A/S436F, A437G, K540E/K540N, A581G and S613A); Mozambique (N=1; NRNIF-ISGEAA); Indonesia (N=1, IRNIS-ISGEAA); frequencies greater than 10% are bolded

Genotypes *	Bangladesh	Myanmar	Thailand	Laos	Cambodia	Vietnam	PNG	Colombia	Peru
NCSIS-ISAKAA	-	-	-	5.2	0.4	-	-	6.7	5.9
NCSIS-IS**G**KAA	-	-	-	-	-	-	-	-	-
NCSIS-I**A**AKAA	-	-	-	-	-	-	-	-	-
NCSIS-I**AG**KAA	-	-	-	-	-	-	-	-	-
NC**N**IS-ISAKAA	-	-	-	-	-	-	-	**60.0**	**52.9**
N**RN**IS-ISAKAA	2.9	-	0.1	**19.6**	1.1	-	9.6	-	-
N**RN**IS-IS**G**KAA	-	-	-	7.2	1.1	-	2.1	-	-
N**RN**IS-IS**GE**AA	8.8	-	-	-	0.3	-	4.3	-	-
N**RN**IS-I**A**AKAA	-	-	-	-	0.4	-	-	-	-
N**RN**IS-I**AG**KAA	-	-	-	1.0	1.5	0.7	-	-	-
N**RN**IS-I**AGE**AA	**17.6**	2.3	1.9	6.2	0.3	-	-	-	-
N**RN**I**F**-ISAKAA	-	-	-	-	-	-	**48.9**	-	-
N**RN**I**F**-IS**G**KAA	-	-	-	-	-	-	7.4	-	-
N**RN**I**F**-IS**GE**AA	-	-	-	-	-	-	**27.7**	-	-
N**RNL**S-IS**GEG**A	2.9	0.5	2.3	-	0.1	-	-	-	-
N**RNL**S-I**AGE**AA	5.9	**10.2**	1.2	-	-	-	-	-	-
**I**C**N**IS-ISAKAA	-	-	-	-	-	-	-	**20.0**	-
**I**C**N**IS-IS**G**KAA	-	-	-	-	-	-	-	6.7	-
**I**C**N**IS-IS**GE**AA	-	-	-	-	-	-	-	-	-
**I**C**N**IS-IS**GEG**A	-	-	-	-	-	-	-	-	**17.6**
**IRN**IS-ISAKAA	-	-	-	**21.6**	4.9	**14.7**	-	-	-
**IRN**IS-IS**G**KAA	5.9	-	-	**16.5**	4.5	9.3	-	-	-
**IRN**IS-IS**G**K**G**A	-	-	0.4	-	0.6	1.3	-	-	-
**IRN**IS-IS**GNG**A	-	1.9	0.4	2.1	8.3	6.0	-	-	-
**IRN**IS-IS**GE**AA	2.9	2.8	0.3	6.2	4.4	9.3	-	-	-
**IRN**IS-IS**GEG**A	-	6.0	6.7	1.0	1.0	3.3	-	-	-
**IRN**IS-I**A**AKAA	-	-	-	-	0.8	2.0	-	-	-
**IRN**IS-I**AG**KAA	-	-	-	7.2	5.6	**22.0**	-	-	-
**IRN**IS-I**AG**KA**S**	-	-	-	-	-	-	-	-	-
**IRN**IS-I**AGE**AA	**20.6**	4.7	5.0	3.1	**15.6**	3.3	-	-	-
**IRN**IS-**VAG**KAA	-	-	-	-	-	-	-	-	-
**IRN**IS-**VAG**K**GS**	-	-	-	-	-	-	-	-	-
**IRNL**S-IS**GNG**A	-	2.8	6.0	-	**30.7**	0.7	-	-	-
**IRNL**S-IS**GE**AA	2.9	0.5	1.2	-	2.0	0.7	-	-	-
**IRNL**S-IS**GEG**A	5.9	**34.0**	**60.8**	-	1.8	-	-	-	-
**IRNL**S-I**FG**KAA	-	-	-	-	-	-	-	-	-
**IRNL**S-I**FGE**A**S**	-	-	-	-	0.3	**16.0**	-	-	-
**IRNL**S-I**AGE**AA	**14.7**	**25.1**	9.5	-	**10.0**	2.7	-	-	-
