# Supplementary material for: Genetic diversity of the Plasmodium falciparum GTP-cyclohydrolase 1, dihydrofolate reductase and dihydropteroate synthetase genes reveals new insights into sulfadoxine-pyrimethamine antimalarial drug resistance
Source: PLoS Genet. 2020 Dec 31;16(12):e1009268. doi: 10.1371/journal.pgen.1009268 (PMC7774857; doi:10.1371/journal.pgen.1009268)
Supplement: S3 Table — (PDF) [file pgen.1009268.s006.pdf]

S3 Table. Frequency (%) of *pfgch1* amplifications (DupA-J) by country.

Country	Region	n	A	B	C	D	E	F *	G **	H ***	I +	I ++	I +++	J
Mauritania	WAfr	55	-	-	-	-	-	-	-	-	1.8	1.8	**10.9**	-
Mali	WAfr	258	-	-	-	-	-	-	-	0.4	5.4	2.3	4.3	-
Senegal	WAfr	76	-	-	-	-	-	-	-	-	6.6	6.6	-	-
Gambia	WAfr	167	-	-	-	-	-	-	-	0.6	4.8	3.0	0.6	-
Guinea	WAfr	99	-	-	-	-	-	-	-	-	**10.1**	**16.2**	5.1	-
Burkina Faso	WAfr	11	-	-	-	-	-	-	-	-	-	-	-	-
Ivory Coast	WAfr	46	-	-	-	-	-	-	2.2	4.3	2.2	4.3	**10.9**	-
Ghana	WAfr	468	-	-	-	-	0.9	0.6	1.7	0.6	3.0	3.8	**18.4**	0.2
Benin	WAfr	61	-	-	-	-	-	-	-	-	1.6	1.6	4.9	-
Nigeria	WAfr	13	-	-	-	-	-	-	-	-	**15.4**	-	**30.8**	-
Cameroon	CAfr	158	-	-	-	-	2.5	-	-	7.6	6.3	3.8	1.3	-
DRC	CAfr	179	-	-	-	-	1.1	-	-	-	**10.1**	**19.0**	0.6	-
Uganda	EAfr	4	-	-	-	-	-	-	-	-	**25.0**	**25.0**	-	-
Kenya	EAfr	50	-	-	6.0	-	-	-	-	-	**46.0**	**20.0**	2.0	-
Tanzania	EAfr	216	-	-	-	-	-	-	-	-	**46.8**	**18.1**	-	-
Malawi	SAfr	215	-	-	-	-	-	-	-	-	**64.2**	**29.3**	-	-
Madagascar	SAfr	18	-	-	-	**11.1**	-	-	-	-	-	-	-	-
Ethiopia	HoAfr	22	-	9.1	-	-	-	-	-	-	-	-	-	-
Bangladesh	SEA	34	-	-	-	-	2.9	**17.6**	-	-	-	-	-	-
Myanmar	SEA	215	-	-	-	-	**17.2**	**14.0**	3.3	0.5	-	-	-	-
Thailand	SEA	684	-	-	-	-	**27.6**	9.1	2.2	-	-	-	-	-
Laos	SEA	97	-	-	-	-	-	-	1.0	-	-	-	-	-
Cambodia	SEA	710	0.4	-	0.3	-	7.2	-	2.7	-	-	-	-	-
Vietnam	SEA	150	-	-	-	-	0.7	-	**11.3**	-	-	-	-	-

* GB4, T996; ** KH02; *** NF54; + KE01; ++ KE01/ML01; +++ ML01; DRC = Democratic Republic of Congo; PNG = Papua New Guinea; gDupA - H are gene amplifications; pDupI (3 types) and pDupJ are promoter amplifications; omitted are Mozambique (n=1; J type) and Indonesia (n=1, no amplification); Papua New Guinea (N=94), Colombia (N=15) and Peru (N=17) have no amplifications; WAfr West Africa; CAfr Central Africa; EAfr East Africa; SAfr Southern Africa; HoAfr Horn of Africa; SEA South(east) Asia; frequencies greater than 10% are bolded
