# Supplementary material for: Genetic diversity of the Plasmodium falciparum GTP-cyclohydrolase 1, dihydrofolate reductase and dihydropteroate synthetase genes reveals new insights into sulfadoxine-pyrimethamine antimalarial drug resistance
Source: PLoS Genet. 2020 Dec 31;16(12):e1009268. doi: 10.1371/journal.pgen.1009268 (PMC7774857; doi:10.1371/journal.pgen.1009268)
Supplement: S4 Table — (PDF) [file pgen.1009268.s007.pdf]

S4 Table. The main *pfgch1* amplifications with the most frequent *pfdhfr/pfdhps* genotypes in Africa and Asia (%).

Duplication	N	Genotypes *	Number of genotypes	West Africa	Central Africa	East Africa	Southern Africa	Horn of Africa	Southeast Asia
A	3	NCSIS-ISAKAA	3	-	-	-	-	-	**100**
B	2	**IRN**IS-IS**GE**AA	2	-	-	-	-	**100**	-
C	5	**IRN**IS-IS**G**KAA	2	-	-	-	-	-	**40**
		**IRNL**S-I**FG**KAA	3	-	-	**60**	-	-	-
D	2	**IRN**IS-IS**G**K**G**A	2	-	-	-	**100**	-	-
E	289	**IRN**IS-IS**GEG**A	20	-	-	-	-	-	6.9
		**IRN**IS-I**AGE**AA	36	-	-	-	-	-	**12.5**
		**IRNL**S-IS**GNG**A	16	-	-	-	-	-	5.5
		**IRNL**S-IS**GEG**A	124	-	-	-	-	-	**42.9**
		**IRNL**S-I**AGE**AA	45	-	-	-	-	-	**15.6**
F *	101	N**RN**IS-I**AGE**AA	7	-	-	-	-	-	6.9
		**IRN**IS-IS**GEG**A	10	-	-	-	-	-	9.9
		**IRNL**S-IS**GEG**A	49	-	-	-	-	-	**48.5**
		**IRNL**S-I**AGE**AA	11	-	-	-	-	-	**10.9**
G **	68	**IRN**IS-IS**G**KAA	5	4.4	-	-	-	-	2.9
		**IRNL**S-IS**GNG**A	16	-	-	-	-	-	**23.5**
		**IRNL**S-IS**GEG**A	13	-	-	-	-	-	**19.1**
		**IRNL**S-I**FGE**A**S**	17	-	-	-	-	-	**25.0**
H ***	20	**IRN**IS-IS**G**KAA	10	**15.0**	**35.0**	-	-	-	-
		**IRN**IS-**VAG**KAA	2	-	**10.0**	-	-	-	-
		**IRN**IS-**VAG**K**GS**	3	**10.0**	5.0	-	-	-	-
I +	347	**IRN**IS-IS**G**KAA	43	7.8	4.6	-	-	-	-
		**IRN**IS-IS**GE**AA	214	-	-	**23.1**	**38.6**	-	-
		**IRN**IS-IS**GEG**A	30	-	0.3	8.1	0.3	-	-
I ++	207	**IRN**IS-IS**G**KAA	46	8.2	**14.0**	-	-	-	-
		**IRN**IS-IS**GE**AA	88	0.5	-	**12.6**	**29.5**	-	-
		**IRN**IS-IS**GEG**A	18	-	0.5	8.2	-	-	-
		**IRN**IS-I**AG**KAA	18	7.2	1.4	-	-	-	-
I +++	125	NCSIS-IS**G**KAA	8	6.4	-	-	-	-	-
		**IRN**IS-IS**G**KAA	39	**30.4**	0.8	-	-	-	-
		**IRN**IS-I**A**AKAA	7	5.6	-	-	-	-	-
		**IRN**IS-I**AG**KAA	32	**24.8**	0.8	-	-	-	-
		**IRN**IS-I**AG**KA**S**	9	7.2	-	-	-	-	-

* GB4, T996; ** KH02; *** NF54; + KE01; ++ KE01/ML01; +++ ML01; gDupA - H are gene amplifications; pDupI (3 types) and pDupJ are promoter amplifications; * based on *pfdhfr* (N51I, C59R, S108N, I164L and I306F) and *pfdhps* (I431V, S436A, A437G, K540E/K540N, A581G and A613S); frequencies of at least 10% are bolded
